# Socioeconomic factors, perceived stress, and social support effect on neonatal nurse burnout in China: a cross-sectional study

**DOI:** 10.1186/s12912-023-01380-z

**Published:** 2023-06-26

**Authors:** Zhen-peng Huang, Fang Huang, Qun Liang, Feng-zhen Liao, Chuan-zhuang Tang, Min-lan Luo, Si-lan Lu, Jing-jing Lian, Shan-e Li, Su-qiao Wei, Bin Wu

**Affiliations:** 1 Faculty of Nursing, Guangxi University of Chinese Medicine, Nanning, China; 2 Department of Neonatology, Nanning Maternal and Child Health Hospital, Nanning, China; 3 Pediatric Intensive Care Unit, Nanning Maternal and Child Health Hospital, Nanning, China; 4 Department of Pediatrics, Nanning Second People’s Hospital, Nanning, China

**Keywords:** Socioeconomic factors, Perceived stress, Social Support, Burnout, Neonatal nurse

## Abstract

**Background:**

Neonatal nurses’ working environments are highly stressful, and burnout is common. This study examines the effect of socioeconomic factors, perceived stress, and social support on neonatal nurse burnout.

**Methods:**

A total of 311 neonatal nurses participated in this study. They were administered a validated Maslach Burnout Inventory. The study employed a 14-item perceived stress scale (PSS-14) and a social support rate scale (SSRS) to examine stress, socioeconomic factors, and lifestyles.

**Results:**

Of the neonatal nurses, 40.19% had burnout, 89.60% had mild burnout, and 10.40% had moderate burnout; no neonatal nurse experienced severe burnout. Young nurses and those with low technical skills, poor interpersonal relationships, irregular diet, and insufficient rest were exposed to burnout (all p < 0.05).Most burnout nurses experienced moderate-severe perceived stress, and their PSS-14 scores were higher (all p < 0.05).The scores for objective social support, subjective social support, utilization of social support, total SSRS scores, and the level of social support were all lower in burnout nurses (all p < 0.05). Perceived stress was correlated positively and significantly with emotional exhaustion and personal accomplishment (all p < 0.05). Social support correlated significantly with and reduced personal accomplishments (p < 0.05). Age, poor interpersonal relationships, perceived stress, and social support were all independent factors associated with neonatal nurse burnout (all p < 0.05).

**Conclusion:**

The prevalence of burnout in neonatal nurses was higher than average. Socioeconomic factors, higher perceived stress, and lower social support contribute to neonatal nurse burnout. Nursing managers should pay attention to socioeconomic factors, perceived stress, and social support among neonatal nurses and employ strategies to reduce neonatal nurse burnout.

## Introduction

Nurses play a key part as healthcare workers in the health system. Nursing is a highly stressful occupation; nurses do not only deliver healthcare but also participate in patients’ rehabilitation with limited resources and increasing responsibilities in working environments [1–3]. For example, neonatal nurses must take care of newborns whose physiological functions are frail and have difficulty in adapting to the changing environment, which entails more responsibilities and increases stress [4]. Such an imbalance between increasing nursing responsibilities and coping with stressful working environments can commonly lead to burnout [5].

Globally, there is a high incidence of burnout. Burnout is often defined as a syndrome that results from prolonged job stress, including the dimensions of emotional exhaustion, depersonalization, and reduced personal accomplishment [6]. Burnout mainly leads to loss of motivation, reduces commitment to work, and results in significant consequences for nurses, hospitals, and patients [7]. Burnout is also viewed as one of the most important issues facing various occupations and has been identified as a serious problem for nurses in many countries [8–10].

Previous studies have suggested that 20.90% and 16.60–30% of nurses experience burnout in Europe in America, respectively. Additionally, the prevalence rate of nurse burnout is 56% and 35.50–50% in Japan and China, respectively [11–15]. However, the prevalence of burnout among neonatal nurses remains unknown.

Burnout is associated with the work environment, work-related stress, and job satisfaction, and is related to such factors as lifestyle as interpersonal conflicts among healthcare workers [16–18]. Poor interpersonal relationships and marital status would increase nurses’ vulnerability to burnout [19, 20]. According to the Conservation of Resources (COR) theory, social support loss is a critical component of stress. When resources are lost or no adequate resources are gained, individuals’ negative stress would increase [21]. Recent studies have suggested that being in a state of stress for a prolonged period of time leads to negative psychological outcomes and impacts burnout [21–23]. Studies also confirmed that perceived stress and social support would work on burnout. In stressful working conditions, in particular, social support can help nurses reduce psychological distress, thus impacting burnout [24–27]. However, the impact of socioeconomic factors, perceived stress, and social support on nurse burnout, especially in neonatal nurses, is still unknown.

This study aimed to explore socioeconomic factors such as interpersonal relationships, marital status, perceived stress, and social support, and their impact on neonatal nurse burnout in China.

## Materials and methods

### Samples and study process

Questionnaires were administered using a cross-sectional study. Neonatal nurses were recruited from 11 affiliated university hospitals and 10 primary hospitals in Nanning City through snowball sampling strategy. The questionnaires were distributed to head nurses in neonatology; subsequently, in neonatology, who subsequently distributed the questionnaires to neonatal nurses from May 1 to June 30, 2022. Participants were allowed to take the anonymous questionnaire survey anytime; it was a one-time activity and did not require more than 30 min to complete. Informed consent was obtained from all participants prior to participation in this study. The study was approved by the Institutional Ethical Committee of the Nanning Maternal and Child Health Hospital. All methods in this study were carried out in accordance with relevant guidelines and regulations according to the Declaration of Helsinki.

### Measures and questionnaires

All participants were administered a validated Chinese version of the Maslach Burnout Inventory (MBI), it has developed by Maslach in 1981 and translated into the Chinese version by Li, to measure neonatal nurse burnout [28, 29]. The MBI was measured on three subscales: emotional exhaustion, depersonalization, and personal accomplishment. It included 5, 4, and 6 items in each subscale, respectively. Row scores from 0 to 6 in each item represented a frequency of “never” to “every day” over the course of nearly one year. Researchers calculated each subscale’s item and total scores, with higher scores indicating higher levels of burnout. MBI standard scores were indicated by Y = int (1.33x). Total scores over 50 points were regarded as burnout, 50–74 points were divided into mild burnout, 74–100 points were divided into moderate burnout, and > 100 points were regarded as severe burnout [30]. Cronbach’s alpha of the Chinese version of the MBI was 0.883 [31].

The Chinese version of the 14-item perceived stress scale (PSS-14) was used to detect participants’ perceived stress. The PSS-14 was developed by Cohen et al. in 1983 and has been translated into the Chinese version by Chu [32–34]. Scores from 0 to 4 in each item indicated a frequency of “never” to “every day” over the course of nearly one month. Each item’s score of the PSS-14was calculated. A score of 14–28 points indicated mild perceived stress, 29–42 points indicated moderate perceived stress, 43–56 points indicated moderate-severe perceived stress, and 57 or above points indicated severe perceived stress. Cronbach’s alpha of the Chinese version of the PSS-14 was0.84 [35].

The social support rate scale (SSRS), it has developed by Xiao in 1986, was used to test the participants’ social support. It differentiates the level of social support by measuring three components: objective social support, subjective social support, and the utilization of social support. Items’ scores from each component of the SSRS were calculated. An overall sum below 20 points indicated low-level social support, 20–29 points indicated medium-level social support, and above 30 points indicated high-level social support. The higher the SSRS score, the higher level of social support [36, 37]. Cronbach’s alpha was 0.84.

Demographic features including gender and age were collected. Social feature data were self-reported by all participants as follows: educational level, characteristics of hospitals, professional title, years of work, continuing education, whether or not participants were head nurses, level of satisfaction with interpersonal relationships, previous work experience in anti-COVID-19, marital status, spouse’s occupation, number of children, and whether or not participants had supportive parents. Lifestyle data were self-reported by all participants, as follows in the questionnaire: smoking, alcoholism, irregular diet, and rest.

Cronbach’s alpha for the questionnaires were 0.89; thus, the instrument’s internal consistency reliability coefficient was reliable.

### Analysis on statistics

Statistical analyses were performed using the IBM SPSS Statistics version 25.0. Continuous variables were presented as mean ± standard deviation. Categorical variables were expressed as proportions and percentages. The association between relevant factors and study outcomes was presented as odds ratios (OR) and 95% confidence intervals (95% C.I.). Comparisons of continuous variables were analyzed using the Student’s *t*-test. Comparisons of categorical variables were analyzed on a chi-squared, or *x*^2^, test, Fisher’s exact test, or the appropriate *Ridit* test. Correlation analysis was carried out between perceived stress and social support in burnout. Independent factors associated with neonatal nurse burnout were analyzed using multivariate logistic regression. Statistical significance was set at *P* < 0.05.

## Results

### Neonatal nurse burnout prevalence

According to the formula of sample size estimation used in medical research, the average prevalence of neonatal nurse burnout in China varied from 35.50%to50% [14, 15]. Therefore, the studied required a sample size of at least 281 participants.

A total of 321 neonatal nurses filled out the questionnaires; however, 10 samples were invalid owing to incomplete questionnaires. As such, a total of 311 neonatal nurses were enrolled in the study: 2.89% (9) were male and 97.11% (302) were female; age varied from 20 to 52 (31.36 ± 5.60).

Neonatal nurses often suffered from burnout. Among all neonatal nurses who experienced burnout, 40.19% (125) had burnout, 89.60% (112) had mild burnout, 10.40% (13) had moderate burnout, and none experienced severe burnout.

### Demographic and socioeconomic factors and neonatal nurse burnout

Various demographic factors such as age, socioeconomic factors, such as professional title, and interpersonal relationships all have a significant effect on neonatal nurse burnout. In this study, age, professional title, and interpersonal relationships were significantly associated with burnout syndrome (all *P* < 0.05) (Table 1).

**Table 1:** Demographic and socioeconomic factors among neonatal nurses in burnout

Variables	Category	Nurses in Burnout (%)	Nurses without Burnout (%)	
Gender	Male	5 (4.00)	4 (2.15)	
	Female	120 (96.00)	182 (97.85)	*x*^2^ = 0.37, *P* = 0.54
Age *	20–29 years	65 (52.00)	66 (35.48)	
	30–39 years	52 (41.60)	94 (50.54)	
	40–49 years	8 (6.40)	25 (13.44)	
	Above 50 years	0 (0)	1 (0.54)	*x*^2^ = 3.16, *P* < 0.01
	Mean age	30.16 ± 4.79	32.17 ± 5.97	*t* = 3.15, *P* < 0.01
Educational level	High school or below	1 (0.80)	3 (1.62)	
	Junior college	29 (23.20)	34 (18.28)	
	Undergraduate	95 (76.00)	149 (80.28)	*x*^2^ = 0.81, *P* = 0.42
Hospitals characteristics	Affiliated hospital of universities	81 (64.80)	104 (55.19)	
	Primary hospitals	44 (35.20)	82 (44.09)	*x*^2^ = 2.45, *P* = 0.12
Professional title	Junior	94 (75.20)	112 (60.22)	
	Intermediate	29 (23.20)	68 (36.56)	
	Senior	2 (1.60)	6 (3.22)	*x*^2^ = 7.58, *P* = 0.02
Years of working	1 year below	3 (2.40)	8 (4.30)	
	1–5 years	40 (32.00)	49 (26.34)	
	6–10 years	45 (36.00)	55 (29.57)	
	10 years above	37 (29.60)	74 (39.78)	*x*^2^ = 1.35, *P* = 0.18
Taking Continuing Education		42 (22.58)	74 (59.20)	*x*^2^ = 1.22, *P* = 0.27
Head Nurse		4 (3.20)	10 (5.38)	*x*^2^ = 0.40, *P* = 0.53
Interpersonal relationship	Dissatisfaction	7 (5.60)	3 (1.61)	
	On average	70 (56.00)	67 (36.02)	
	Satisfaction	48 (38.40)	116 (62.37)	*x*^2^ = 18.61, *P* < 0.01
Previous work experience in anti-COVID-19		49 (39.20)	78 (41.94)	*x*^2^ = 0.23, *P* = 0.63
Marital status	Single	46 (36.80)	61 (33.15)	
	Married	77 (61.60)	121 (65.76)	
	Divorced	2 (1.60)	2 (1.09)	*x*^2^ = 0.56, *P* = 0.45
Spouse occupation	Health Care Profession	30 (38.96)	36 (29.75)	
	Non-health Care Profession	47 (61.04)	85 (70.25)	*x*^2^ = 1.80, *P* = 0.18
Numbers of Children	Nullipara	65 (4.8)	72 (38.71)	
	One Child	31 (24.80)	58 (31.18)	
	Two Children	28 (22.40)	54 (29.03)	
	Three or More Children	1 (0.80)	2 (1.08)	*x*^2^ = 5.37, *P* = 0.15
Support parents		91 (72.80)	145 (77.96)	*x*^2^ = 1.58, *P* = 0.45

* all *P* < 0.01

### Lifestyle effect on neonatal nurse burnout

Our study also confirmed that lifestyle impacts neonatal burnout. Irregular diet and rest were much more common among participants who experienced burnout (*P* < 0.05). Alcoholism was also common among neonatal nurses who experienced burnout; however, there was no significant difference due to low prevalence for statistical analyses (*P* > 0.05) (Table 2).

**Table 2:** Lifestyles among neonatal nurses in burnout

Variables	Nurses in Burnout (%)	Nurses without Burnout (%)	
Smoking	2 (1.6)	4 (2.15)	*x*^2^ = 0.12, *P* = 0.73
Alcohol	7 (5.60)	4 (2.15)	*x*^2^ = 1.69, *P* = 0.19
Irregular diet and rest	78 (62.40)	92 (49.46)	*x*^2^ = 5.05, *P* = 0.02

### Perceived stress and neonatal nurse burnout

Among neonatal nurses who experienced burnout, most suffered from moderate to severe perceived stress; however, most neonatal nurses without burnout experienced mild and moderate perceived stress (*P* < 0.05). In addition, the scores of the PSS-14 in neonatal nurses who experienced burnout were higher than those in neonatal nurses without burnout (42.20 ± 5.60 vs. 35.77 ± 5.60, *P* < 0.05) (Table 3).

**Table 3:** Prevalence of perceived stress among neonatal nurses in burnout * (%)

Variables	Nurses in Burnout	Nurses without Burnout
Mild level perceived stress	3 (2.40)	23 (12.37)
Moderate level perceived stress	51 (40.80)	134 (72.04)
Moderate-severe level perceived stress	70 (56.00)	29 (15.59)
Severe level perceived stress	1 (0.80)	0 (0)

* *x*^2^ = 7.67, *P* < 0.01

### Social support and neonatal nurse burnout

Social support significantly impacted neonatal nurse burnout. The scores of objective social support, subjective social support, utilization of social support, and total social support were all lower in neonatal nurses who did show burnout than in those without burnout (all *P* < 0.05) (Table 4). Moreover, the level of social support of neonatal nurses with burnout was lower than that of neonatal nurses without burnout (*P* < 0.05) (Table 5).

**Table 4:** Prevalence of social support among neonatal nurses in burnout

Variables	Nurses in Burnout	Nurses without Burnout	
Objective social support	7.59 ± 2.72	9.44 ± 3.00	*t* = 5.62, P < 0.01
Subjective social support	20.08 ± 5.02	23.04 ± 5.38	*t* = 4.96, P < 0.01
Utilization of social support	7.10 ± 1.69	8.27 ± 1.97	*t* = 5.48, P < 0.01
Total social support scores	34.79 ± 7.15	40.84 ± 8.02	*t* = 6.97, P < 0.01

**Table 5:** Level of social support among neonatal nurses in burnout * (%)

Variables	Nurses in Burnout	Nurses without Burnout
Low-level social support	2 (1.60)	1 (0.54)
Medium-level social support	37 (29.60)	21 (11.29)
High-level social support	86 (68.80)	164 (88.17)

* *x*^2^ = 17.03, *P* < 0.01

### Correlation between perceived stress, social support, and neonatal nurse burnout

Perceived stress and social support factored into the level of burnout among neonatal nurses. Perceived stress was positively correlated with emotional exhaustion, personal accomplishment, and general burnout (all *P* < 0.05). In addition, social support was negatively correlated with personal accomplishments and general burnout (*P* < 0.05) (Table 6).

**Table 6:** Correlation between perceived stress and social support in burnout among neonatal nurses

Burnout Variables	Perceived Stress	Social Support
Emotional exhaustion	*r* = 0.16, *P* = 0.07	*r*=-0.13, *P* = 0.16
Depersonalization	*r*=-0.05, *P* = 0.61	*r*=-0.10, *P* = 0.26
Personal accomplishment	*r* = 0.32, *P* < 0.01	*r*=-0.27, *P* < 0.01
General burnout	*r* = 0.28, *P* < 0.01	*r*=-0.27, *P* < 0.01

### Relevant independent factors for neonatal nurse burnout

Results with statistical significance in previous analysis were further carried out through multivariate logistic regression. Age (OR = 0.49, 95% C.I. 0.31–0.76), interpersonal relationships (OR = 0.04, 95% C.I. 0.37–0.96), perceived stress (OR = 5.33, 95% C.I. 3.15-9.00), and social support (OR = 0.49, 95% C.I. 0.25–0.97) were defined as independent factors associated with neonatal nurse burnout (all *P* < 0.05). Moreover, professional title (*P* = 0.05) may also be an independent factor for neonatal nurse burnout; further research is needed to confirm this (Table 7).

**Table 7:** Multivariate logistic regression analyses on selected factors associated with burnout among neonatal nurses

Selected factors	B	S.E.	Wald	df	Sig.	Exp (B)	95%C.I. for Exp (B)
Age	-0.72	0.23	10.11	1	< 0.01	0.49	(0.31, 0.76)
Technical level	-0.38	0.20	3.84	1	0.05	0.68	(0.47, 1.00)
Interpersonal relationship	-0.52	0.25	4.46	1	0.04	0.59	(0.37, 0.96)
Irregular diet and rest	-0.08	0.28	0.08	1	0.78	0.92	(0.53, 1.60)
Perceived stress	1.67	0.27	39.02	1	< 0.01	5.33	(3.15, 9.00)
Social Support	-0.71	0.35	4.24	1	0.04	0.49	(0.25, 0.97)

These results suggest that older age, better interpersonal relationships, and high-level social support are independent protective factors for burnout among neonatal nurses; however, high perceived stress is an independent risk factor for neonatal nurse burnout.

## Discussion

The COR theory has suggested that people make efforts to obtain, retain, and protect individual resources, such as self-esteem, interpersonal relationships, and social support [38]. Psychological stress occurs when individuals lose or fail to gain resources or are threatened [21]. Typically, stress resulting from a threat or loss of individual resources would induce both revoltive psychological and physical outcomes [21, 39].

A complicated and critical working environment is highly demanding, complicated, and stressful, and high level of workforce stress can easily induce burnout syndrome among nurses, thereby decreasing their job satisfaction and increasing their intent to leave the nursing profession [40, 41]. Newborn babies can suffer from a variety of diseases such as neonatal pneumonia and neonatal asphyxia, and their parents can also suffer from perinatal depression and anxiety disorders. As such, increased requests for nursing care have been raised [42–44]. According to the COR theory, a highly stressful working environment results in individual resources loss; thus, neonatal nurses suffer increased negative incidents such as burnout. In this study, we confirmed that 40.19% of neonatal nurses experienced burnout, which is higher than the general prevalence of burnout among Chinese nurses (35.5%), though the burnout was mild. However, generally, nurse burnout syndrome not only has financial repercussions for the healthcare system but also has a significant impact on nurses’ mental health [45].

Recent studies have confirmed that socioeconomic and lifestyle factors affect burnout syndrome [7, 46]. This study found that neonatal nurses who were young, had a low level of technical skills, broken interpersonal relationships, irregular diets, and lack of rest easily suffered from burnout syndrome. In the early stages of their career, nurses lack experience and strategies to deal with workload and work stress; broken interpersonal relationships would increase stress from job demands and stress [47–49]. Stress would induce both negative psychological and physical effect [21, 39]. It has also been found that higher emotional exhaustion and depersonalization are greatly related to unhealthy lifestyles, and the correlation between burnout and unhealthy behaviors can be defined as an underlying health-impairing process in that work stressors adversely affect nurses’ lifestyles, leading to burnout and health problems [11].

Perceived stress is an outcome variable that measures the experienced stress level as a function of objectively stressful events, coping processes, and personality factors [33]. Individuals with a high level of perceived stress are considered unpredictable, uncontrollable, and overloading life [50]. Higher perceived stress contributes to the incidence of job burnout [51, 52]. In this study, we also found out that PSS-14 scores were higher among neonatal nurses who had burnout, and most of them were perceived to be experiencing moderate-to-severe stress compared to neonatal nurses who were without burnout and had moderate levels of perceived stress. This may be because neonatal nurses were in a highly stressful working condition with significantly limited resources. As the COR theory suggests, individual resources that are lost would aggravate psychological stress and induce revoltive outcomes [21, 39].

Other studies have confirmed that decreased social support increases burnout among nurses [53, 54]. Family and social support are essential for nurses to overcome psychological distress and deal with work stressors, and lack of social support is a crucial risk factor for psychological problems and nurse burnout [55]. Support from family, relatives, friends, and colleagues offers nurses more opportunities to control or avoid negative feelings and prevent burnout syndrome [56]. As the COR theory posits, failure to gain resources would induce psychological stress and would aggravate revoltive physical outcomes [39, 57]. For neonatal nurses with burnout syndrome, objective social support, subjective social support, and utilization of social support were all lower than for health nurses, which would promote the development of burnout syndrome.

This study has confirmed that perceived stress is positively associated with emotional exhaustion and personal accomplishment, while social support is negatively correlated with personal accomplishment. It has been suggested that higher perceived stress and lower social support promote the development of and increase burnout syndromes.

This study had several limitations. First, the self-report questionnaire survey may have garnered a few deviations or false information from the participants in the study. Second, this study was conducted in Nanning City, resulting in a limited sample size. Additionally, because this study was a cross-sectional study, no mediation and/or moderation analysis was carried out; thus, further research is required.

In conclusion, this study confirms that the burnout prevalence of neonatal nurses is high. Socioeconomic factors play an important role in burnout among neonatal nurses as do higher perceived stress and lower social support. Nursing managers should care for neonatal nurses’ mental health and devise strategies and policies to reduce neonatal nurse burnout.

## Data Availability

The data that support the findings of this study are available from the corresponding author upon reasonable request.

## Abbreviations

COR theory: Conservation of Resources (COR) Theory
MBI: Maslach Burnout Inventory
PSS-14: 14-item Perceived Stress Scale
SSRS: Social Support Rate Scale
OR: Odds Ratios
95% C.I.: 95% Confidence Intervals
